# The complexity of mental health care for people with COPD: a qualitative study of clinicians’ perspectives

**DOI:** 10.1038/s41533-021-00252-w

**Published:** 2021-07-22

**Authors:** Juliet Wang, Karen Willis, Elizabeth Barson, Natasha Smallwood

**Affiliations:** 1 Melbourne Medical School, The University of Melbourne, Parkville, VIC Australia; 2 School of Allied Health, Human Services and Sport, La Trobe University, Melbourne, VIC Australia; 3 Division of Critical Care and Investigative Services, Royal Melbourne Hospital, Parkville, VIC Australia; 4 Allied Health Department, Royal Melbourne Hospital, Parkville, VIC Australia; 5 Department of Respiratory & Sleep Medicine, Royal Melbourne Hospital & The Alfred Hospital, Melbourne, VIC Australia; 6 Department of Immunology & Respiratory Medicine, Monash University, Melbourne, VIC Australia

**Keywords:** Chronic obstructive pulmonary disease, Health care

## Abstract

Anxiety and depression are common mental health illnesses in people with chronic obstructive pulmonary disease (COPD). However, patients often decline formal mental health care with barriers identified at the patient, health provider and health system levels. Currently clinicians’ perspectives on this issue are not well understood. A qualitative study using semi-structured interviews was undertaken to explore clinician perceived barriers and facilitators to acceptance of psychological care amongst people with COPD. Twenty-four Australian respiratory health professionals participated. Interview transcripts were analysed thematically. An overarching theme of ‘complexity’ was identified, which was evident across five domains: (1) physical and mental health illnesses; (2) psychosocial circumstances; (3) community views and stigma; (4) educational needs and knowledge gaps; (5) navigating the health system. Targeted patient education around psychological interventions and integration of mental health clinicians within multidisciplinary outpatient respiratory services are needed to address the current challenges.

## Introduction

Chronic obstructive pulmonary disease (COPD) is a highly prevalent, progressive condition leading to significant morbidity and mortality worldwide^1–3^. In Australia, COPD is the third greatest contributor to overall disease burden, and fifth most common cause of death^4^. In the United States of America, more than 15.7 million Americans have been diagnosed with COPD, with 96% of individuals living with at least one other chronic health condition^5,6^.

Importantly, depression is the second most common self-reported comorbidity amongst people with COPD^6^. The mean prevalence of depression and anxiety in individuals with COPD are estimated to be 40% (range 8–80%) and 36% (range 6–74%), respectively^7,8^. Furthermore, the prevalence of panic disorder is approximately ten times higher in people with COPD than in the general population^9^. Mental health issues in COPD are associated with reduced ability to cope with physical aspects of the illness^10,11^, poorer quality of life, lower pulmonary function^12,13^, more frequent hospital admissions with longer length of stay, reduced medication compliance and lower adherence to rehabilitation and other treatment regimens^10,14–18^.

While the Global Initiative for Chronic Obstructive Lung Disease guidelines recommend actively screening for and managing psychological comorbidities in COPD^11,16,19^, reportedly less than one-third of people with COPD and mental illness receive adequate treatment for psychological issues^16,20^. Absence of a standardised approach to the assessment and treatment of mental health conditions in people with COPD may partly explain why anxiety and depressive disorders are under-treated^13,16,21^. However, the under-treatment of mental health disorders in people with COPD is most likely due to a series of complex inter-related factors, including patient, provider and health system barriers^16,22^. Patient-derived barriers to mental health treatments include: stigma related to mental health, inadequate mental health appraisal or recognition and difficulty accessing care^12^. Additional barriers at the provider and health system levels may include: clinicians’ lack of confidence in completing mental health assessments, failure to screen for psychological issues, poor inter-professional communication and inadequate insurance coverage of mental health treatments^16,23^. Crucially, there are limited empirical data demonstrating the effects of these barriers on the uptake of mental health care in COPD, particularly when referrals to psychological or pharmacological therapies have been initiated by respiratory clinicians^24^.

Clinicians’ perspectives on patients’ mental health needs and their experiences providing mental health care to people with COPD are not well understood. It has been suggested that clinicians’ perceptions of patients’ emotional and psychological needs are closely linked to their preparedness to offer ancillary support services such as pulmonary rehabilitation (PR)^25,26^. Furthermore, health professionals’ prioritisation of these issues can directly impact patients’ willingness to accept psychological support services^25,27^. A better understanding of clinicians’ perspectives would help to delineate potential barriers to care and provide strategies for improving access to psychological care for people with COPD. Therefore, this study aimed to explore respiratory clinicians’ perspectives of mental health illnesses in COPD, their attitudes surrounding provision of mental health care and their understanding of the barriers and facilitators to acceptance of psychological care by people with COPD.

## Results

### Participant characteristics

Twenty-eight health professionals were invited to participate in the study and 24 agreed. The remaining four did not respond after repeat follow-up emails were sent and were not contacted further. Twenty-four respiratory health professionals (17 females, 7 males), ranging in age from 31 to 64 years, from various professions were recruited. Participants worked within public and/or private hospitals or were based at community health centres (Table 1). There were no participant withdrawals from the study.

**Table 1:** Participants’ characteristics (*n* = 24).

Characteristic	Count and frequency
*Gender*	
Male	7 (29.2%)
Female	17 (70.8%)
*Age*^a^	50.5 (44.3–55.8)
*Years in practice*^a^	19.5 (12.5–25.8)
*Occupation*	
Respiratory physician	12 (50.0%)
Nurse	5 (20.8%)
Physiotherapist	5 (20.8%)
Social worker	1 (4.2%)
Psychologist	1 (4.2%)
*Type(s) of practice*	
Public practice only	9 (37.5%)
Private practice only	1 (4.2%)
Public and private practice	7 (29.2%)
Community centre	7 (29.25)
*Number of patients with COPD seen/week*^a^	15 (5–20)

*COPD* chronic obstructive pulmonary disease.

^a^Median (interquartile range).

### Complexity in mental health care for patients with COPD

An overarching theme of ‘complexity’ was identified, which was evident across five domains: (1) physical and mental health illnesses; (2) psychosocial circumstances; (3) community views and stigma; (4) educational needs and knowledge gaps; and (5) navigating the health system. Within each domain, health professionals acknowledged that a number of patient, provider and health system factors affected the uptake of mental health care. Participants identified the need to integrate psychological supports into routine respiratory care to facilitate better mental health care engagement. Tables 2–6 summarise the resulting sub-themes and illustrative quotes.

**Table 2:** Complex physical and mental health illnesses.

Subtheme	Quotes
Mental health issues are prevalent and linked to COPD severity	“In chronic disease, they [mental health illnesses] are relatively common and in COPD probably particularly common. Especially as you get to severe COPD, extremely common.” (HP5, Respiratory physician, male)
Mental health issues are part of multi-morbidity	“They often have multiple, multisystem diseases and a lot of things that interact. So I think it’s just the selected population that I would see will often have a lot of medical problems, and with that a lot of psychiatric issues.” (HP7, Respiratory physician, male)
Differentiating mental health issues from physical symptoms	“Sometimes it can be difficult to disentangle their somatic symptoms of their breathlessness on exertion, and anxiety. But the two interplay.” (HP3, Respiratory physician, male)
Complex, chronic mental health issues	“Some of them [COPD patients] might be schizophrenic, some of them might have PTSD, some of them might have childhood abuse, so there’s other groups of people that we find really hard to help because their problems have been very long-standing and whatever’s been offered has probably already been offered to them before in the past.” (HP2, Community centre nurse, female)

**Table 3:** Psychosocial circumstances.

Subtheme	Quotes
Complex social circumstances	“Panicky, breathlessness, distressing childhood, PTSD, difficult life, lots of psychosocial factors, living in a difficult economic environment. Having difficult family situations, sexual abuse, poverty, a million different…. so many different triggers I suppose. Current smoking, ex-smoker, feeling depressed about their continued smoking, unable to stop smoking.” (HP9, Respiratory physician, female)
Financial cost as a barrier to mental health care	“The financial barrier is a problem with these healthcare visits to psychologists, I know when they have the mental health care plan, they have a certain number of visits every year but there’s still an out-of-pocket [cost]. I have some patients who are literally on the bread line, and even though they’re in an affluent area, they’re still in housing commission [public housing]. That’s a big barrier for them financially, because if they have to pay an out-of-pocket expense to see a psychologist, versus putting food on the table for that week, you can see what’s going to win.” (HP13, Community centre nurse, female) “There’s been limitations in how they can access the treatment; often cost-related as well. And caught up in the fact that a lot of the patients with bad COPD often come from low socioeconomic backgrounds and they’re not working, there’s often huge financial strains on them.” (HP7, Respiratory physician, male)
Relationship between smoking, COPD and mental health	“It’s probably the other way around – chicken versus egg – usually their significant mental health problem precedes their COPD, and probably contributes to it because they smoke so much.” (HP15, Respiratory physician, female) “It allays their anxiety – the continuing to smoke, and of course the continuing to smoke makes their lung disease worse and worse, so it’s a vicious circle.” (HP9, Respiratory physician, female) “There’s a group of patients who are still smoking and that’s a really difficult thing to manage as well…. it prevents people from necessarily seeking treatment or engaging in treatment.” (HP7, Respiratory physician, male)

**Table 4:** Community views and stigma.

Subtheme	Quotes
Stigma of smoking	“Smoking is the new leprosy in our society, and people feel ashamed and often condemned and kind of get no sympathy because ‘you did it to yourself’ and ‘you’re an evil smoker’ and some people are continuing to smoke so I think that is a big barrier to people seeking any kind of services as well.” (HP22, Social worker, female) “That addictive process that has happened over many years – you can’t blame the patient for that. But they often do themselves, and they think ‘if only I hadn’t smoked then I wouldn’t be in this situation, life would be so much better and that’s making me feel pretty sad.’” (HP5, Respiratory physician, male)
Stigma of COPD	“There is definitely a stigma associated with COPD. I mean, sometimes they want the label of “asthma” because that’s more socially acceptable than COPD.” (HP6, Respiratory physician, male) “They feel a bit guilty about it, and I think that hinders their openness sometimes – because they feel that it’s been self-inflicted, self-caused, and they are often the ones responsible for what’s happened to them, and of course you must never ever be pejorative about that.” (HP5, Respiratory physician, male)
Stigma of mental health	“There’s kind of the old perception that if we start talking about mental health issues then that means they’re ‘crazy’. Or they’ve got schizophrenia, or bipolar. And they’re really reluctant to talk about their issues because they don’t want to be labelled, so there’s that element of reluctance to seek assistance.” (HP22, Social worker, female) “They [antidepressants] are perceived as mind-altering drugs, and taking one of those drugs is perceived as a sign that you have a mental health problem…. mental health problems are always associated with stigma.” (HP3, Respiratory physician, male) “I think there’s a stigma. They’re happy to say, ‘I’m feeling a bit depressed but no, I’m not going to see a psychologist cos I don’t need it.’ Psychologist no, psychiatrist no, medications no. Some of them are already on medications for depression, but they say ‘no, I don’t need it’. They may not know what they’re taking but they’ll say it’s for my “mood”. So, a lot of people like to refer it as my “mood”, but not the word depression.” (HP2, Physiotherapist, female)

**Table 5:** Educational needs and knowledge gaps.

Subtheme	Quotes
The need for education about mental health illnesses and supports	“A lot of the times they say, ‘oh I don’t believe in depression, it’s all mind over matter’. So that’s just an unhealthy inbuilt belief that they’ve had all their lives. I suppose the only way to deal with that is through education, and making them understand it is a disease just as much as anything else is, and it’s not just a physical disease, it’s a mental disease. And how it can negatively impact on their physical health as well as their mental health. So a lot of it is about education.” (HP21, Community centre nurse, female)
HPs identifying patients’ mental health issues and attempting to manage them	“One of the standard questions is that ‘in the last month or so, have you felt feelings of helplessness or feeling depressed?’ So, we do ask them that. All of our patients are screened with the HADS form. And if the screening is high, we talk to them about their feelings; it gives us permission to talk to them about it. We say to them, ‘looking at this, you scored quite high, can we discuss that?’” (HP2, Physiotherapist, female) “In terms of intervention, [patients’ mental health care] would sit with a psychiatrist or a psychologist. Because certainly that’s not my area of expertise. But identifying that is something I can do, in terms of identifying if patients are struggling with their mental health.” (HP23, Public hospital nurse, female) “There’s a lot we can do, not only physically, but also attempt to support in their wellbeing. I spend a lot of my time reassuring them; I try to give them strategies to improve their quality of life each day; I’d encourage mobility and exercise, all of the other non-pharmacological strategies; pulmonary rehabilitation, certainly give them a sense of much better wellbeing, and I do support them as much as I can.” (HP5, Respiratory physician, male)
HPs providing education and support through ongoing relationships	“It takes a lot of hard work in terms of building that rapport. And when you get to that spot, it’s absolutely wonderful because you can see how vulnerable and scared they are. And you can offer help, so it’s not something you develop straight way.” (HP21, Community centre nurse, female) *“*I think more recognition and referral, and even just having conversations about acknowledging and letting patients acknowledge and realise that their physical and mental health conditions probably impact each other…. I guess that’s important knowledge for individuals in that situation; so maybe just to be able to cope with it better…. or if they’re feeling unwell mentally that that’s managed.” (HP10, Respiratory physician, female)
HPs limited training and expertise in mental health care	“It’s quite limited, any training you might have in counselling communication skills, unless you’re specifically targeted or enrolled in a particular course that was directed towards that. But in general I don’t think I have a lot of training in you know, managing mental health issues. A lot of it is learnt as you go.” (HP14, Respiratory physician, female)

*HP* health professional, *HADS* Hospital Anxiety and Depression Scale.

**Table 6:** Complexity navigating the health system.

Subtheme	Quotes
Navigation is complex for HPs and patients	“The mental health system is…bizarre. It’s so hard to understand how to navigate mental health services. I think if I’m saying that as a health practitioner, I can’t imagine how hard it is for a client…. And even if we can get people on a mental health care plan, you know they can’t always access timely appropriate psychological services or psychologists. It’s just really difficult.” (HP13, Community centre nurse, female) “We find that even if you can detect [mental health illnesses], we’re often short of options of knowing what we can do about it, apart from primarily linking back to their GP [family physician] as their first and foremost point of contact. So I think we’ve often found that it’s a case of you can notice it, but you feel a bit powerless to actually do much about it.” (HP16, Physiotherapist, male)
Fragmented care	“Patients often don’t want to go somewhere else, I guess that’s the other thing – we have such fragmented care, that they’ll see the doctor at the clinic and then they come down to pulmonary rehab and see us, and then they have to go back to their GP to get a care plan to see someone else, somewhere else. So I think if we could have a better multidisciplinary team to manage all those issues, I think that would work better.” (HP19, Physiotherapist, female)
Limited access to care	“Easier access to both psychology and palliative care services. Well the access is there, but it’s just the waiting lists are a problem etc. And availability. Perhaps a more defined pathway of how to – and tools to manage anxiety, would be helpful.” (HP4, Respiratory physician, female) “A well-rounded multidisciplinary team that included a psychologist in each department would be fabulous; easier access for the patient. Cos you’ve got that net around; if you’ve got a good multidisciplinary team where the respiratory physician, the physio, the psychologist, whoever else is involved in the care of that person – can talk freely and hold them as a team – then that’s going to increase the capacity to do a better job.” (HP24, Private psychologist, female)
Time constraints	“It’s incredibly difficult in a public outpatient setting to ever have any time to come close to addressing any mental health issues; almost other than to acknowledge them. Because you’ve usually got a 15-minute window to sort out active medical problems…. So, all conversations are quite limited or potentially limited anyway.” (HP10, Respiratory physician, female)
Need for standardised approach to mental health	“One of the problems with a lot of these services is that they’re very ad-hoc and there’s a lot of local variation in how these services are delivered, so unless you understand that….you can’t access them for the patients. So, I think it’d be really helpful if there was… as well as *more…* there was a more standardized service to improve accessibility.” (HP12, Respiratory physician, male)

*HP* health professional, *GP* general practitioner (family physician).

#### Physical and mental health illnesses

Most participants perceived that mental health issues were more prevalent in people with COPD than in other long-term conditions (Table 2), and this perspective was consistent across the different professions. Participants noted that mental health issues were linked to COPD severity, multi-morbidity, increased frequency of hospital admissions, respiratory exacerbations and poorer physical health status overall. In addition, participants acknowledged that while anxiety and depression were common in people with respiratory disease, other mental health conditions such as chronic schizophrenia, post-traumatic stress disorder (PTSD) and substance addiction were also over-represented in people with COPD.

One participant expressed a divergent view that mental health conditions were over-diagnosed in this patient group. This participant, whose background was in private psychology, believed that patients’ psychological distress could be interpreted as a grief reaction to their COPD, rather than a separate mental health diagnosis. It is highly likely that this participant’s professional background influenced their assessment of mental illness prevalence in COPD. This participant maintained that psychological distress was a common feature of those affected by COPD, and cautioned against over-pathologising mental health symptoms, suggesting that proper referencing of diagnostic criteria would help to differentiate those with existing psychopathology from those with an adjustment disorder to their respiratory illness. In keeping with the perspectives of the other participants, this clinician agreed that people with COPD faced strong emotional reactions to the physical and functional limitations of their chronic respiratory disease, and typically required psychological support and input.

All participants regardless of age, background and years of practice were cognisant of the complex interrelationship between patients’ physical and psychological conditions. Participants perceived that patients’ physical decline had a profound impact on their mental health, and vice versa. Major contributors to patients’ psychological distress included patients’ experiences of the breathlessness-anxiety cycle, fear of death and dying and functional impairment. Most participants recognised the importance of addressing patients’ physical and mental health conditions concurrently. However, several indicated that the overlap between patients’ physical and mental health symptoms sometimes made detection of psychological issues more challenging.

#### Psychosocial circumstances

Many participants described difficulties in managing mental health issues in people with COPD due to their complex psychosocial circumstances (Table 3). They discussed how patients whose lives were ‘more fraught’ with psychosocial issues, such as unemployment, addiction, financial challenges and social isolation, were less likely to prioritise or utilise mental health care. Some participants suggested that substance use, including continued smoking, despite respiratory disease progression could indicate patients’ general disengagement with health care. Several participants identified an association between smoking, COPD and mental health issues. Furthermore, patients who continued smoking despite having COPD were noted to delay seeking help, and have poorer engagement with physical and mental health treatments.

#### Community views and stigma

Participants suggested that complex community views regarding mental health, smoking and COPD all contributed to poor uptake of mental health care by people with COPD. Patients were described as experiencing two layers of stigma, comprising shame or embarrassment from having a smoking-related illness such as COPD, and stigma due to mental health illness and treatments (Table 4). Participants observed that patients tended to internalise negative societal attitudes towards smoking, which then contributed to psychological distress. The belief that COPD was a self-inflicted disease was sometimes expressed to patients by other health care providers. One participant indicated that patients’ guilt around smoking tended to impact on their openness during consultations, therefore highlighting the importance of minimising blame in COPD. Moreover, several participants indicated that patients held negative perceptions of mental health and this contributed to reluctance in engaging with mental health care. These multiple layers of stigma related to smoking, COPD and mental health issues, when combined, were challenging to address.

#### Educational needs and knowledge gaps

The need for further education and knowledge about mental health was an important theme discussed by participants (Table 5). Participants reported that patients displayed variable awareness of their psychological symptoms, but most had limited knowledge of available mental health treatments and supports. Similarly, while all participants were cognisant of the mental health struggles of people with COPD, and many felt proficient in identifying these issues, only a few displayed confidence in adequately managing patients’ mental health issues. Several participants admitted to a lack of knowledge, training and expertise in providing such supports themselves. Participants who had received some education in psychological techniques had acquired these skills through self-directed learning, research pursuits, or experiential learning. A few participants described utilising ongoing therapeutic relationships with patients to make observations on mental health, provide psychoeducation and encourage acceptance of mental health care.

#### Navigating the health system

Several participants discussed how navigating the current mental health system was difficult because of system-level barriers and time constraints (Table 6). A lack of coordinated care, limited availability of mental health services, overbooked clinics and long waiting lists often resulted in instances of fragmented care. Many health professionals also mentioned a lack of direct referral pathways or integrated psychology in respiratory clinics, which prevented the coordination of mental health services for patients. While several participants discussed the role of the family physician in coordinating mental health supports, they also suggested that primary care providers faced similar limitations regarding time constraints and access to mental health services. Overall, participants cited the need for a standardised approach to mental health care, and considered that involving mental health clinicians in multidisciplinary respiratory services would help to overcome challenges in navigating the mental health system.

## Discussion

This qualitative study revealed respiratory clinicians’ beliefs regarding the mental health needs of people with COPD and clinician perceived barriers and facilitators to acceptance of psychological care. Our study expands on previous research^16,23^, by examining the barriers to mental health care, and provides new insights into the effects of fragmented care, psychosocial complexity, and community stigma on patients’ engagement with mental health treatments.

Complexity was a core theme across our findings, which was reflected in participants’ understanding of the long-standing nature of patients’ mental health illnesses, as well as the challenges of providing psychological support to an already vulnerable patient group. Several participants identified a degree of complexity around caring for people with both pre-existing psychological morbidity and anxiety and depression linked to having COPD. While much of the literature is focused objectively on the high rates of clinical depression or anxiety in this patient group^28,29^, there appears to be less discussion of the implications of pre-morbid mood disorders on patients’ physical and psychological health in COPD.

Despite assumptions to the contrary from previous research^11,16,30^, an important and unique finding in this study was that respiratory clinicians recognised the complex mental health needs of people with COPD, and proactively attempted to manage psychological issues. Participants described routinely enquiring about patients’ mental health or using validated screening tools to detect psychological symptoms. This is in contrast to other studies that have suggested respiratory clinicians under-recognise emotional distress, believe psychological illnesses fall outside their clinical practice or minimise the need for mental health care altogether^11,23,31–33^. These contrasting findings may partly be explained by study design, as previous studies have used surveys, service-use questionnaires or group discussions to gauge clinicians’ awareness of mental health issues in COPD^16,23,34^. By contrast the qualitative nature of our study allowed participants to describe in detail their clinical practice regarding addressing patients’ mental health needs, as well as the barriers and enablers to such care.

Major barriers to the provision of mental health care included health professionals’ time constraints, limited access to psychological services and difficulties navigating a complex mental health system. Similar to other studies^16,23,35^, participants described difficulties addressing patients’ psychological issues during short respiratory consultations, with many clinicians emphasising the need for more time to discuss complex issues and to build a therapeutic relationship. Participants also believed that current health care funding models prioritised high patient throughput, and did not facilitate access to psychological services. Several participants raised cost as a barrier to uptake of mental health treatments in Australia. Similarly, previous studies have suggested patients view financial and insurance issues as key barriers to utilising mental health treatments^35,36^.

Contextual factors, including complex social circumstances and stigma associated with smoking-related conditions like COPD, have been identified in our study and previous research as contributing to under-utilisation of mental health care^10,23,37,38^. Participants perceived patients’ mental health issues to be closely intertwined with a long history of heavy smoking, despite having severe symptoms from COPD. Similar findings in a pilot study of a psychology-led smoking cessation programme have shown that patients use smoking as a way to regulate negative emotions^38,39^, and those who were unable to reduce or quit smoking reported adverse life experiences such as a history of trauma or childhood abuse^37,39^. In addition, participants in our study suggested that people with COPD face a ‘double stigma’ related to both COPD and mental illness, which impacts upon engagement with psychological therapies. Whereas previous research has shown that the ‘stigma of psychology’ prevents patients’ accessing mental health treatments^30,35,39^, our study suggests that double-layered stigma acts as a unique barrier to service utilisation amongst this group of patients.

Our findings are consistent with previous research identifying a lack of universal mental health training across the health workforce as one reason for the insufficient uptake of mental health care^40^. Our results provide a strong rationale for the inclusion of mental health clinicians within multidisciplinary respiratory services (hospital outpatient or PR programmes) to address these issues. Other clinicians who can support patients with COPD and mental illness include clinical nurse consultants and social workers who have received specific training in mental health care. Ideally these clinicians would be supported by clinical psychologists and psychiatrists. Earlier studies have proposed that supporting the localisation of psychological services within rehabilitation units can enable knowledge-transfer between clinicians, while also encouraging treatment teams to routinely consider the mental health of people with COPD alongside their physical health^40–42^. Therefore, a focus around mental health education and capacity-building within the multidisciplinary team may help to address knowledge gaps amongst respiratory clinicians, and aid in broadening the role of mental health care provision to those with other skillsets. Importantly, embedding psychological services within respiratory care will improve patient access to an otherwise inaccessible and complex mental health system.

Earlier studies have proposed that PR programmes also represent an ideal opportunity to integrate more collaborative care between mental health specialists and the multidisciplinary respiratory team^16,17,37,41^. However, empirical data demonstrating the inclusion of psychological services within PR programmes are scarce^39^. A working model of integrated psychological care across respiratory inpatient, outpatient and group settings has proposed that psychologists play an important role in supplementing the core self-management strategies of PR, as well as increasing patients’ insights into the psychological consequences of their chronic lung disease^39^. Of the emerging evidence, a 9-month trial integrating clinical psychology into a specialist respiratory department in the United Kingdom has shown promising results^43^. This intervention produced improved clinical outcomes for patients with depression or anxiety, and was well received by patients and staff alike^43^. Further research regarding the economic feasibility and clinical impact of models of care, which incorporate psychological services, is warranted.

Participants from the current study also identified the need for a standardised approach to mental health care to overcome barriers such as limited access to trained therapists and fragmented care. An embedded collaborative care model using shared mental health assessment tools and guided by mental health specialists could enable more streamlined screening and treatment pathways^16^. Moreover, a recent review of cognitive behavioural therapy for the treatment of anxiety and depression in COPD also recommended that patients have access to mental health treatments within standard outpatient care to mitigate stigma associated with seeking external help^11,30^. Incorporating mental health clinicians within respiratory settings might also facilitate more equitable psychology access for ‘hard to reach’ patients^39^, thereby eliminating some of the financial and logistical barriers to mental health care mentioned in this study. Future studies should evaluate the effectiveness of these integrated care models in improving patients’ access to and uptake of mental health supports.

While several complex, inter-related factors have been identified as contributing to the under-utilisation of mental health care by people with COPD, our findings may not be generalisable to other populations or geographical settings. Nevertheless, purposive sampling allowed for the inclusion of a diverse range of clinician views. Moreover, due to the impact of COVID-19, only health professionals’ perspectives of patients’ subjective experiences of mental health care could be obtained. Future research should clarify COPD patients’ and their caregivers’ perspectives regarding mental health needs.

While health professionals were cognisant of the high levels of psychological distress experienced by people with COPD and were willing to manage comorbid mental health conditions, a number of perceived patient, provider and health system barriers prevented patients’ uptake of recommended treatments. Clinicians identified a clear need for increased patient education and support regarding mental health illnesses in COPD, alongside more mental health training and inter-professional collaboration between clinicians involved in the care of people with COPD. Collaborative care models that facilitate the integration of mental health clinicians into routine respiratory outpatient care or PR are likely to enable greater patient access to mental health treatments and are worthy of exploration.

## Methods

### Design, setting and recruitment

An exploratory qualitative design was used to understand respiratory clinicians’ perspectives of managing mental health issues in COPD, specifically the perceived barriers and facilitators to patients’ uptake of mental health care. The Consolidated Criteria for Reporting Qualitative Research, a validated checklist for appraisal of qualitative studies, was used for reporting^44^.

Purposive sampling was used to recruit a broad sample of health professionals (respiratory clinicians, physiotherapists, nurses and other allied health staff), balanced according to gender and diversity of experience. Recruited participants included people working within public and/or private hospital respiratory departments or affiliated community services in the Australian states of Victoria and South Australia. Participants were initially informed of the study via email and invited to participate by the study’s lead investigator (N. S.). Individuals who expressed interest in participating were then contacted by the project interviewer (J. W.) within a week, provided with a participation information statement and informed of the consent and withdrawal processes. All participants were required to complete a consent form prior to participation. Ethical and governance approval for this study was obtained from the Melbourne Health Human Research Ethics Committee (Ref: 2019.281).

### Data collection

Demographic data including clinicians’ occupations and duration of clinical practice were collected at the commencement of in-depth semi-structured interviews. The mean length of interviews was 40–45 min, with one or two interviews of a shorter duration (20–22 min) due to participants’ time constraints and travelling commitments. Interviews were conducted by a single interviewer (J. W.) between February and May 2020 and were undertaken by phone or face-to-face. An interview topic guide facilitated discussions relating to participants’ perspectives of mental health care in the context of COPD (see Supplementary Appendix). Participants were asked to reflect on their experiences of identifying and managing mental health issues in COPD, as well as to describe any barriers and facilitators to uptake of mental health care. Interviews were audio-recorded, de-identified and transcribed verbatim. Field notes of the participants’ key observations were recorded by the interviewer during and promptly after interviews, then added to transcripts. Participants were informed that they could freely access and edit copies of their interview transcripts upon request. During the study, no transcripts were returned for comment or correction. No repeat interviews were conducted.

### Analysis

Demographic data are reported using counts and frequencies. A descriptive, thematic analysis, following Braun and Clarke^45^, was undertaken. Thematic analysis aims to synthesise ideas from participants’ interviews and provide a framework for understanding recurring concepts that constitute broader themes^46^. These themes help to link individual experiences with the more general insights that are evident from the data and are used to explain or interpret social phenomena^46^. This method of qualitative analysis has the benefit of providing a theoretically flexible approach to interpreting data across a range of research questions, and can generate unanticipated but highly nuanced insights^45^.

Transcripts were coded line-by-line that involved manually highlighting sections of the transcript, assigning labels to each new concept and idea, and ensuring codes were reflective of intrinsic meanings within the data. Key labels and concepts were then iteratively evaluated by each of the authors through multiple rounds of coding, which allowed for refinement of similar codes and identification of common patterns across the data. Intercoder reliability was encouraged by ensuring at least two members of the research team coded each of the transcripts independently and agreed upon the final coding used^46^. Codes were subsequently extrapolated to overarching themes encapsulating the richness of the data. Analysis occurred concurrently with data collection to determine thematic saturation, that is, until no new concepts or themes emerged. Themes were reviewed and discussed by all authors at regular meetings to ensure transparency and consistency across the analysis process, as well as to reach consensus on the final core themes of the study.

At the time the study was undertaken, the first author (J. W.) was a final-year medical student, trained and supervised in qualitative methods by an experienced qualitative researcher (K. W.). J. W. had no working relationships with any of the participants. The second author (K. W.) is a qualitative researcher with extensive experience in health research. The third author (E. B.) is a clinical psychologist with insights into multidisciplinary respiratory and mental health services at a major public hospital. The senior author (N. S.) is a consultant respiratory physician and academic with an in-depth knowledge of respiratory practices across Australia and internationally. Together, all authors contributed diverse insights into caring for or observing people with COPD in their illness experience. Arguably this approach is advantageous, as previous studies have indicated that involving a team of researchers with differing backgrounds aids in improving the breadth and depth of analysis and subsequent findings^46^. Any potential biases or subjective idiosyncrasies that may have arisen^47^ were addressed through regular meetings, in which all authors exchanged ideas, compared conclusions and concurred on final themes of the study.

### Reporting summary

Further information on research design is available in the Nature Research Reporting Summary linked to this article.

## Supplementary information

Reporting Summary

Supplementary Information

## Data Availability

The data analysed in this study are available from the corresponding author upon reasonable request.
