# Supplementary material for: The complexity of mental health care for people with COPD: a qualitative study of clinicians’ perspectives
Source: NPJ Prim Care Respir Med. 2021 Jul 22;31:40. doi: 10.1038/s41533-021-00252-w (PMC8298614; doi:10.1038/s41533-021-00252-w)
Supplement: Supplementary file 1 — Reporting Summary [file 41533_2021_252_MOESM1_ESM.pdf]

nature research

Corresponding author(s): A/Prof Natasha Smallwood

Last updated by author(s): Jun 5, 2021

Reporting Summary

Nature Research wishes to improve the reproducibility of the work that we publish. This form provides structure for consistency and transparency in reporting. For further information on Nature Research policies, see our Editorial Policies and the Editorial Policy Checklist.

Statistics

For all statistical analyses, confirm that the following items are present in the figure legend, table legend, main text, or Methods section.

n/a	Confirmed	
☒	☐	The exact sample size (n) for each experimental group/condition, given as a discrete number and unit of measurement
☒	☐	A statement on whether measurements were taken from distinct samples or whether the same sample was measured repeatedly
☒	☐	The statistical test(s) used AND whether they are one- or two-sided<br>*Only common tests should be described solely by name; describe more complex techniques in the Methods section.*
☒	☐	A description of all covariates tested
☒	☐	A description of any assumptions or corrections, such as tests of normality and adjustment for multiple comparisons
☒	☐	A full description of the statistical parameters including central tendency (e.g. means) or other basic estimates (e.g. regression coefficient) AND variation (e.g. standard deviation) or associated estimates of uncertainty (e.g. confidence intervals)
☒	☐	For null hypothesis testing, the test statistic (e.g. F, t, r) with confidence intervals, effect sizes, degrees of freedom and P value noted<br>*Give P values as exact values whenever suitable.*
☒	☐	For Bayesian analysis, information on the choice of priors and Markov chain Monte Carlo settings
☒	☐	For hierarchical and complex designs, identification of the appropriate level for tests and full reporting of outcomes
☒	☐	Estimates of effect sizes (e.g. Cohen's d, Pearson's r), indicating how they were calculated

Our web collection on statistics for biologists contains articles on many of the points above.

Software and code

Policy information about availability of computer code

Data collection	No software was used for data collection
Data analysis	N/A

For manuscripts utilizing custom algorithms or software that are central to the research but not yet described in published literature, software must be made available to editors and reviewers. We strongly encourage code deposition in a community repository (e.g. GitHub). See the Nature Research guidelines for submitting code & software for further information.

Data

Policy information about availability of data

All manuscripts must include a data availability statement. This statement should provide the following information, where applicable:

- Accession codes, unique identifiers, or web links for publicly available datasets
- A list of figures that have associated raw data
- A description of any restrictions on data availability

The data analysed in this study are available from the corresponding author upon reasonable request.

Field-specific reporting

Please select the one below that is the best fit for your research. If you are not sure, read the appropriate sections before making your selection.

☐ Life sciences ☒ Behavioural & social sciences ☐ Ecological, evolutionary & environmental sciences

For a reference copy of the document with all sections, see nature.com/documents/nr-reporting-summary-flat.pdf

Behavioural & social sciences study design

All studies must disclose on these points even when the disclosure is negative.

Study description	An exploratory qualitative design was used to understand respiratory clinicians' perspectives of managing mental health issues in COPD, specifically the perceived barriers and facilitators to patients' uptake of mental health care
Research sample	Twenty-eight health professionals were invited to participate in the study and twenty-four agreed. The remaining four did not respond after repeat follow-up emails were sent and were not contacted further. Twenty-four respiratory health professionals (17 females, 7 males), ranging in age from 31 years to 64 years, from various professions were recruited. Participants worked within public and/or private hospitals or were based at community health centres.
Sampling strategy	Purposive sampling was used to recruit a broad sample of health professionals (respiratory clinicians, physiotherapists, nurses and other allied health staff), balanced according to gender and diversity of experience.<br><br>Qualitative analysis occurred concurrently with data collection to determine thematic saturation, that is, until no new concepts or themes emerged.
Data collection	Interviews were audio-recorded using a simple audio recording device, de-identified, and transcribed verbatim using Microsoft Word. Interviews were conducted by a single researcher over the phone or face-to-face with participants. There were no other individuals present besides the participant and the researcher at the time of interviews.
Timing	Interviews were conducted by a single interviewer between February and May 2020.
Data exclusions	No data were excluded from analysis.
Non-participation	Twenty-eight health professionals were invited to participate in the study and twenty-four agreed. The remaining four did not respond after repeat follow-up emails were sent and were not contacted further. Twenty-four respiratory health professionals (17 females, 7 males), ranging in age from 31 years to 64 years, from various professions were recruited. There were no participant withdrawals from the study.
Randomization	N/A

Reporting for specific materials, systems and methods

We require information from authors about some types of materials, experimental systems and methods used in many studies. Here, indicate whether each material, system or method listed is relevant to your study. If you are not sure if a list item applies to your research, read the appropriate section before selecting a response.

Materials & experimental systems

n/a	Involved in the study	
☒	☐	Antibodies
☒	☐	Eukaryotic cell lines
☒	☐	Palaeontology and archaeology
☒	☐	Animals and other organisms
☐	☒	Human research participants
☒	☐	Clinical data
☒	☐	Dual use research of concern

Methods

n/a	Involved in the study	
☒	☐	ChIP-seq
☒	☐	Flow cytometry
☒	☐	MRI-based neuroimaging

Human research participants

Policy information about studies involving human research participants

Population characteristics	Twenty-four respiratory health professionals (17 females, 7 males), ranging in age from 31 years to 64 years, from various professions were recruited. Participants worked within public and/or private hospitals or were based at community health centres.

Recruitment	A broad sample of health professionals (respiratory clinicians, physiotherapists, nurses and other allied health staff) were recruited through public and/or private hospitals and Victorian HARP Respiratory Services. Participants were initially informed of the study via email and invited to participate by the study's lead investigator. Individuals who expressed interest in participating were then contacted by the project interviewer within a week, provided with a participation information statement, and informed of the consent and withdrawal processes.
Ethics oversight	Melbourne Health Human Research Ethics Committee (Ref: 2019.281)

Note that full information on the approval of the study protocol must also be provided in the manuscript.
