# Supplementary material for: The complexity of mental health care for people with COPD: a qualitative study of clinicians’ perspectives
Source: NPJ Prim Care Respir Med. 2021 Jul 22;31:40. doi: 10.1038/s41533-021-00252-w (PMC8298614; doi:10.1038/s41533-021-00252-w)
Supplement: Supplementary file 2 — Supplementary Information [file 41533_2021_252_MOESM2_ESM.pdf]

Supplementary Appendix:

Interview topic guide for health professionals

INFORMATION ABOUT YOU?

1. Can you tell me about yourself and your current role?

   (Prompts: Age, gender, discipline (medical specialist/GP/physiotherapist/nurse/other), location of practice (public hospital/general practice/community centre)

2. How long have you been in practice – how long in this role, other roles?

3. What types of patients do you see (eg only chronic disease, specific cultural groups, number of COPD patients seen each week)

PSYCHOLOGICAL ISSUES AND LONG TERM CONDITIONS

4. In your experience how common are psychological / psychosocial problem in patients with chronic disease generally, and COPD, in particular?

5. What types of psychological problems do you typically see in patients with chronic disease generally, and COPD specifically?

PRESENTATION AND DETECTION

6. Do you think patients with COPD recognise when they experience psychological symptoms?
   If so – how do they seem to view their psychological symptoms?

7. Do patients self-report psychological symptoms? (with or without being prompted?)

8. Do you routinely screen for depression or anxiety as part of your management of COPD?

MANAGEMENT

9. Could you tell me about your typical response to a patient who presents with both depression and COPD, anxiety and COPD or all three? (when would treatment be offered; what treatment; referral?)

10. What do you think are the barriers for patients with COPD receiving treatment for depression and/or anxiety? (Prompts – patient lack of prioritisation; health system constraints?)

11. What do you think would assist you to better manage this patient group?

TRAINING AND DEVELOPMENT

12. Of the training you have had, what has been useful for the management of patients with depression and/or anxiety and a long term condition?

Is there anything else you would like to add about the mental health issues faced by patients with COPD, and how we can better assist them? Thank you for your time.
